# Preceding symptoms and temporal development of cortical superficial siderosis in cerebral amyloid angiopathy: a case report

**DOI:** 10.1186/s12883-023-03300-9

**Published:** 2023-06-30

**Authors:** Naja H. Andersen, Rolf A. Blauenfeldt, Ronni Mikkelsen, Claus Z. Simonsen

**Affiliations:** 1 Department of Neurology, Aarhus University Hospital, Aarhus, Denmark; 2 Department of Clinical Medicine, Aarhus University, Aarhus, Denmark; 3 Department of Neuroradiology, Aarhus University Hospital, Aarhus, Denmark

**Keywords:** Amyloid spells, Cortical superficial siderosis, Cerebral amyloid angiopathy, Case report, Brain magnetic resonance imaging

## Abstract

**Background:**

We present a case illustrating evolution of symptoms and brain magnetic resonance imaging in cortical superficial siderosis.

**Case presentation:**

A 74-year-old man with no prior medical history presented with transient focal neurological episodes with subtle imaging changes. There was no evidence of cortical superficial siderosis. Two weeks later, the patient was readmitted with new episodes, and had developed cortical superficial siderosis adjacent to a cerebral microbleed. Transient focal neurological episode secondary to cortical superficial siderosis was diagnosed together with probable cerebral amyloid angiopathy.

**Conclusion:**

Clinical symptoms may precede the development of cortical superficial siderosis prior to being detectable on brain MRI. This case highlights the temporal development of cortical superficial siderosis.

## Background

Cerebral amyloid angiopathy (CAA) is a small- and medium-sized vessel disease affecting arteries in the cerebral cortex and overlying leptomeninges. The disease is caused by a progressive deposition of amyloid in the vessel wall resulting in fragile vessels with a tendency of hemorrhage. The disease is found more frequently with increasing age [1]. Clinically, it can present with lobar intracerebral hemorrhage (ICH), lacunar ischemic stroke, transient focal neurological episodes, cognitive impairment or dementia. Brain magnetic resonance imaging (MRI) can reveal prior lobar ICH, cortical microbleeds, perivascular spaces in the centrum semiovale, white matter hyperintensities in a multi-spot pattern and/or cortical superficial siderosis (cSS). Recently, diagnostic criteria for CAA have been updated making it possible to diagnose probable CAA with higher sensitivity in vivo than previously [2]. Transient focal neurological episodes (known as “amyloid spells”), is a common clinical presentation of patients with CAA, easily mimicking a variety of other conditions, including transient ischemic attack, migraine auras and focal seizures*.* Amyloid spells are believed to reflect cortical irritation caused by cSS which is characterized by deposition of hemosiderin in the subarachnoid space due to breakdown of blood components [3, 4]. The pathophysiologic mechanism behind development and evolution of cSS is incompletely understood but may present discrete bleeding events or blood-leakage from fragile leptomeningeal vessels [5]. The clinical symptoms are likely caused by irritation of the cortex eliciting cortical spreading depression [6].

## Case

A 74-year-old, right-handed man, with no past medical history or vascular comorbidities and no prior head trauma presented with sudden left arm weakness, paresthesia, word finding difficulties and involuntary movements of the fingers. Symptoms remitted after 20 min, except paresthesia. Within the past two month, the patient had experienced multiple transient episodes of left arm paresthesia.

**Fig. 1:**
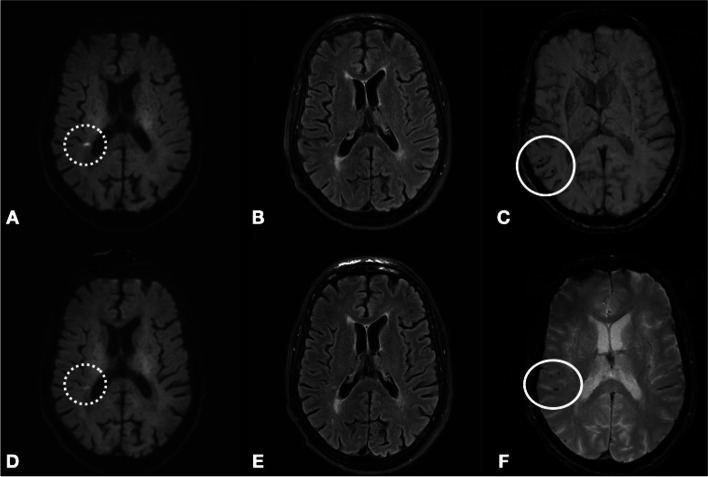
Diffusion-weighted imaging (DWI). Fluid attenuated inversion recorvery (FLAIR). Susceptibility weighted imaging (SWI)/T2* on day 0 (**A**-**C**) and 14 (**D**-**F**). DWI sequence (**A** and **D**) shows a hyperintense lesion in relation to the right lateral ventricle (dotted circle). SWI reveal multiple cortical microbleeds in the same area (solid circle)

The neurological examination was unremarkable. Brain MRI revealed restricted diffusion of the white matter substance in relation to the right lateral ventricle and multiple cortical microbleeds on the susceptibility weighted imaging (SWI) sequence. A cluster of microbleeds could be seen in relation to the right central sulcus (see Fig. 1). Lab results, carotid ultrasound and 7-day Holter monitoring were normal. Apolipoprotein E epsilon 4 allele status was unknown. The patient was diagnosed with transient ischemic attack and dual antiplatelet therapy was prescribed for 3 weeks followed by antiplatelet monotherapy together with a statin.

Eight days later, the patient presented with recurrent paresthesia of the left upper arm and the left side of the face. A second MRI was unchanged (not shown). The symptoms were thought to be caused by recrudescence and the patient was discharged.

**Fig. 2:**
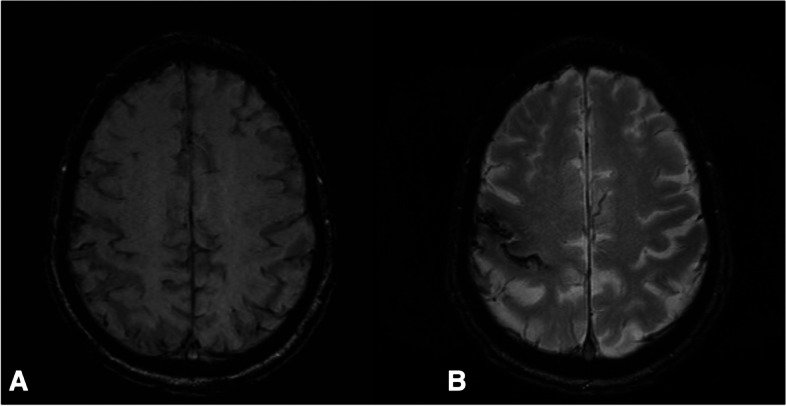
SWI (**A**) and T2* (**B**) at day 0 and 14 respectively. On A there is very faint superficial siderosis in the central sulcus which is much more pronounced at the last examination (**B**)

Fourteen days after the first admission, the patient developed transient slurred speech and weakness in the left arm and face. Duration was approximately 1 h. Neurological examination revealed discrete pronator drift of the left hand and slightly reduced sensibility in the left upper limb. Shortly after admission, the patient developed expressive aphasia, dysarthria, left-sided facial paresis, paresis and reduced sensation and ataxia of the upper limb. A third brain MRI was performed showing that cSS had now developed in the right central sulcus (see Fig. 2). All three MRIs were perfomed on the same 3 Tesla scanner.

Transient focal neurological episode secondary to cSS was diagnosed together with probable CAA. Antiplatelet therapy was discontinued. Attention was drawn to intensive blood pressure management

## Discussion

This case gives insight into clinical symptoms preceding the development of cSS prior to being detectable on brain MRI. The patient had transient symptoms that was ascribed to as a transient ischemic attack where this diagnosis was made more likely due to diffusion restriction in the right periventricular area. Later, cSS developed in the right central sulcus, suggesting that several events may precede actual radiological demonstrable cSS. The diffusion restriction was probably asymptomatic and silent DWI lesions are also described in CAA [1]. The case illustrates the difficulties in navigating between transient ischemic attack, transient focal neurological episodes and recrudescence [7]. The anatomical congruence between a DWI lesion, cluster of cerebral microbleed and later cSS development suggest an ongoing pathological process of blood-leakage or minor bleeds from CAA affected vessels in a fragile region of the brain. This process may have been accelerated by the initial dual antiplatelet therapy in this case. Antithrombotic use in CAA patients presenting with transient focal neurological episodes has been shown to be an independent risk factor for development of ICH [8]. cSS evolution and severity (> 2 sulci) on MRI may be markers of disease severity and risk of future ICH and antiplatelet therapy should be avoided if possible [5].

## Conclusion

We present a case of CAA presenting with transient focal neurological episodes without initial evidence of cSS. Two weeks later the patient presented with new episodes, and had developed cSS adjacent to a cerebral microbleed. Clinical symptoms may precede the development of cSS prior to being detectable on brain MRI.

## Data Availability

All patient material in this case report was collected from the electronic patient journal system, a national repository of record information from all public hospitals in Denmark including Aarhus University Hospital, where the patient was admitted. The corresponding author has full access to all the medical records and imaging examinations that were carried out during the hospitalization at the Department of Neurology, Aarhus University Hospital. The data and imaging used during the current study are available from the corresponding author on reasonable request.

## Abbreviations

CAA: Cerebral amyloid angiopathy
cSS: Cortical superficial siderosis
DWI: Diffusion weighted imaging
FLAIR: Fluid-attenuated inversion recovery
ICH: Intracerebral hemorrhage
MRI: Magnetic resonance imaging
SWI: Susceptibility weighted imaging
